# Supplementary material for: Orthostatic hypotension and neurocognitive disorders in older women: Results from the EPIDOS cohort study
Source: PLoS One. 2023 Feb 24;18(2):e0281634. doi: 10.1371/journal.pone.0281634 (PMC9955614; doi:10.1371/journal.pone.0281634)
Supplement: S1 Table — (DOCX) [file pone.0281634.s001.docx]

**Supplementary Table 1.** Baseline characteristics of 2,276 participants according to the follow-up at 7 years.

|  | Followed at 7-years |  |  |
| --- | --- | --- | --- |
|  | No  (n=2,019) | Yes  **(**n=257**)** | P-value* |
| **Clinical measures** |  |  |  |
| Age (years), mean ± SD | 80.0 ± 3.6 | 79.3 ± 3.2 | **0.002** |
| Body mass index (kg/m^2^) |  |  | 0.389 |
| <21: underweight | 330(16.3) | 47(18.3) |  |
| [21-25[ : Normal | 790(39.1) | 108(42.0) |  |
| [25-30[ : Overweight | 739(36.6) | 80(31.1) |  |
| ≥30: Obesity | 160(7.9) | 22(8.6) |  |
| Number of comorbidities ^†^, mean ± SD | 2.6 ± 1.9 | 3.4 ± 1.4 | **<0.001** |
| Orthostatic hypotension, n (%) | 280(13.9) | 54(21.0) | **0.002** |
| ΔSBP (%), mean ± SD | 0.70 ± 8.52 | -0.36 ± 8.6 | **0.034** |
| ΔDBP (%), mean ± SD | -2.82 ± 11.26 | -1.94 ± 15.30 | 0.776 |
| Pulse pressure, mean ± SD  History of stroke, n (%) | 67.6 ± 15.7  58(2.9) | 68.1 ± 13.0  1(0.4) | 0.522  **0.018** |
| Disability ^‡^, n (%) | 478(23.7) | 22(8.6) | **<0.001** |
| Regular physical activity, n (%) | 1119(55.4) | 133(51.8) | 0.265 |
| Use psychoactive drugs ^\|\|^, n (%) | 831(41.2) | 114(44.4) | 0.327 |
| Use vitamin D supplements, n (%) | 331(16.4) | 47(18.3) | 0.442 |
| DBP: diastolic blood pressure; IADL: Instrumental Activities of Daily Living score; SBP: systolic blood pressure; SD: standard deviation; SPMSQ: Short Portable Mental Status Questionnaire; *: comparisons based on Chi-square test or the Fisher exact test for qualitative variables, and the Student's t-test or the nonparametric Mann-Whitney U test for quantitative variables, as appropriate; ^†^: among hypertension, diabetes, dyslipidemia, coronary heart disease, chronic obstructive pulmonary disease, peripheral vascular disease, cancer, stroke, Parkinson disease and depression; ‡: Instrumental Activities of Daily Living score <7/8; \|\|: benzodiazepines or antidepressants or neuroleptics; P-value significant (i.e. P<0.05) indicated in bold. |  |  |  |
