# Supplementary material for: Orthostatic hypotension and neurocognitive disorders in older women: Results from the EPIDOS cohort study
Source: PLoS One. 2023 Feb 24;18(2):e0281634. doi: 10.1371/journal.pone.0281634 (PMC9955614; doi:10.1371/journal.pone.0281634)
Supplement: Attachment — Submitted filename: Response to Reviewers_Manuscript_Duval_et_al.doc [file pone.0281634.s002.doc]

Manuscript:

- ID#: PONE-D-22-15462
- Entitled: **"** **Orthostatic hypotension and neurocognitive disorders in older women:**
- **Results from the EPIDOS cohort study."**
- Submitted for publication in PLOS ONE

We thank the Reviewer for the review. As suggested, we have performed a revision of our manuscript. All the changes are highlighted in yellow on the revised manuscript. Please, find below our point-by-point responses to the comments offered by the Reviewer.

**Reviewers' comments:

Reviewer's Responses to Questions**

**Question 1:** Is the manuscript technically sound, and do the data support the conclusions?

The manuscript must describe a technically sound piece of scientific research with data that supports the conclusions. Experiments must have been conducted rigorously, with appropriate controls, replication, and sample sizes. The conclusions must be drawn appropriately based on the data presented.

Reviewer #1: Partly

Reviewer #2: Yes

We thank reviewers for these responses.

**Question 2:** Has the statistical analysis been performed appropriately and rigorously?

Reviewer #1: Yes

Reviewer #2: Yes

We thank reviewers for these responses.

**Question 3:** Have the authors made all data underlying the findings in their manuscript fully available?

The [PLOS Data policy](http://www.plosone.org/static/policies.action#sharing) requires authors to make all data underlying the findings described in their manuscript fully available without restriction, with rare exception (please refer to the Data Availability Statement in the manuscript PDF file). The data should be provided as part of the manuscript or its supporting information, or deposited to a public repository. For example, in addition to summary statistics, the data points behind means, medians and variance measures should be available. If there are restrictions on publicly sharing data—e.g. participant privacy or use of data from a third party—those must be specified.

Reviewer #1: No

Reviewer #2: Yes

This is an important question. The data used in this study are from a third party, i.e. from the EPIDOS study, a French observational prospective multicentric national cohort. The public deposit of all the cohort data is subject to the agreement of the investigators of the EPIDOS study.

**Question 4:** Is the manuscript presented in an intelligible fashion and written in standard English?

PLOS ONE does not copyedit accepted manuscripts, so the language in submitted articles must be clear, correct, and unambiguous. Any typographical or grammatical errors should be corrected at revision, so please note any specific errors here.

Reviewer #1: Yes

Reviewer #2: Yes

We thank reviewers for these responses.

**Question 5:** Review Comments to the Author

**Comments of Reviewer #1:**

I reviewed the manuscript entitled “Orthostatic hypotension and neurocognitive disorders in older women: Results from the EPIDOS cohort study.” It is very interesting with a large sample size but has many concerns that need to be considered.

**Comment 1:** All the abbreviations should be defined.

We thank Reviewer #1 for his vigilance. All abbreviations are defined in the revised version of the manuscript.

**Comment 2:** Considering that almost half of the elderly patients use antihypertensive drugs, and the patients taking antihypertensive drugs were also not included in this study, it seems that the results are far from reflecting real life and cannot be generalized.

The Reviewer #1 is correct. We chose to exclude patients taking antihypertensive treatment at baseline since this is a major potential confounding factor in the relationship between orthostatic hypotension and cognitive functioning. However, this choice reduces the representativeness of our results in the general population. This limitation has been described in the discussion of the article. Please, see corrections on page 19.

**Comment 3:** Furthermore, given the fact that hypertensive patients were included in the study, in such patients should have been evaluated with a cut-off of 30 mm Hg fall in SBP, which may be more appropriate, since blood pressure fall after standing up largely depends on baseline BP ( https://pubmed.ncbi.nlm.nih.gov/29562291/ , https://pubmed.ncbi.nlm.nih.gov/30900378/ )

We thank Reviewer #1 for pointing out this important point of method. We took this comment into account and modified the cut-offs to define orthostatic hypotension according to the hypertensive status of the patients in this cohort. The results section and tables have been updated with the new data. These modifications do not change the meaning or the interpretation of our results. The only significant change following these modifications is the appearance of a significant association, not surprisingly, between disability and cognitive diagnosis at 7 years according to model B in Table 3. Please, see correction in Table 1, 2 and 3 on page 10, 12 and 14, supplementary table 1 on page 16, and in the results section on page 9.

**Comment 4:** Literature review in the MN needs to update since many recent studies, in which OH was demonstrated that it may be related to dementia, have been overlooked. (https://pubmed.ncbi.nlm.nih.gov/34255194/ https://pubmed.ncbi.nlm.nih.gov/33975062/ https://pubmed.ncbi.nlm.nih.gov/33735870/ . Please discuss them.

We thank Reviewer #1 for these very interesting references discussed in the Discussion section.

The meta-analysis of Isik et al. shows, very interestingly, a risk multiplied by 2.5 of finding OH in patients with Alzheimer's disease. This reference has been added under number 26.

The second reference of Isik et al. highlights a higher prevalence of OH in patients with Lewy body disease, Alzheimer's disease, and vascular dementia compared to healthy subjects. In this study, the drop of DBP was associated with vascular cognitive disorders, contrary to our study which highlighted a significant relationship between the variation of SBP and cognitive disorders. This reference has been added under number 32.

The last reference of Kocyigit et al. showed interestingly that a better nutritional status seems to improve global cognition and functional abilities in patients with OH, and thus malnutrition could be implicated in the relationship between cognition and orthostatic hypotension in older people. This reference has been added under number 50.

**Comment 5:** All medications that can cause OH in your study population, such as antiparkinson medication, antidepressants, and antipsychotics, should have been evaluated in the study. All these concerns have to be clarified and mentioned as limitation.

The Reviewer #1 is correct. We took into account psychoactive drugs as a potential confounding factor. The details of the patients' psychoactive treatments and the taking of antiparkinsonian treatments were unfortunately not available in the EPIDOS database. Only psychoactive drug use was available in the database. We added this limitation in the Discussion section.

**Comments of Reviewer #2:**

In the manuscript, Duval et al. have reported the relationship between “Orthostatic hypotension and neurocognitive disorders in older women in the EPIDOS cohort study". This study is interesting. However, there were several critical flaws.

Major comments
**Comment 1:** The power analysis
Did the authors perform the power analysis? If not, there was some possibility that the results were derived by chance.

We thank Reviewer #2 for this comment. The power analysis was not performed initially and represents a potential limitation of this study discussed in the Discussion section of the manuscript. Nevertheless, the number of 2,715 subjects studied constitutes a large sample size which remains a strength of this study.

**Comment 2:** n=257
It was complex to undestand how the authors reached the n=257. It would be helpful if there was the flow chart for the way of reaching to n=257.

We thank Reviewer #2 for his proposal to add a flow chart to improve the understanding of the way of reaching to n=257 for analyzes at 7 years of follow-up. Please see the flow chart on Figure 1.

**Comment 3:** Pulse pressure
Pulse pressure might be adjusted in the regression model. How the systolic, diastolic or mean BP was adjusted instead for pulse pressure?

We thank Reviewer #2 for this comment. The pulse pressure was indeed taken into account from the first version of the manuscript as a covariate in the analysis model.

**Comment 4:** Orthostatic hypertension
Did the authors take into account for orthostatic hypertension? This might be confounded in the weak relationship between OH and cognitive dysfunction in the prospective study.

The Reviewer #2 is correct. Orthostatic hypertension was not taken into account as a covariate in this study, which may represent a limit in taking into account potential confounding factors in the weak relationship between OH and cognitive dysfunction in the prospective study. This limit had been discussed in the Discussion section.

However, the Systolic Blood Pressure changes while standing (ΔSBP) variable takes into account downward variations in SBP during the transition to orthostatism but also increases in SBP to orthostatism.

**Comment 5:** Cognitive decline
How would be the result if the dependent variable was defined as delta cognitive function (Baseline score minus score at 7yrs) instead of cognitive dysfunction?

We thank Reviewer #2 for this comment. Unfortunately, the SPMSQ score was not collected in the database at 7 years. Only cognitive status was available.
