# Supplementary material for: Orthostatic hypotension and neurocognitive disorders in older women: Results from the EPIDOS cohort study
Source: PLoS One. 2023 Feb 24;18(2):e0281634. doi: 10.1371/journal.pone.0281634 (PMC9955614; doi:10.1371/journal.pone.0281634)
Supplement: Attachment — Submitted filename: Response 2 to Reviewers_Manuscript_Duval_et_al.rtf [file pone.0281634.s003.rtf]

Manuscript: 
−	ID#: PONE-D-22-15462R2
−	Entitled: " Orthostatic hypotension and neurocognitive disorders in older women:
−	Results from the EPIDOS cohort study."
−	Submitted for publication in PLOS ONE


We thank Reviewers for the second review of our manuscript. As suggested, we have performed a revision of our manuscript. All the changes are highlighted in yellow on the revised manuscript. Please, find below our point-by-point responses to the comments offered by the Reviewer. 

Reviewers' comments:

Question 1. If the authors have adequately addressed your comments raised in a previous round of review and you feel that this manuscript is now acceptable for publication, you may indicate that here to bypass the “Comments to the Author” section, enter your conflict of interest statement in the “Confidential to Editor” section, and submit your "Accept" recommendation.

Reviewer #1: All comments have been addressed

Reviewer #2: All comments have been addressed

We thank reviewers for these responses.


Question 2. Is the manuscript technically sound, and do the data support the conclusions?

The manuscript must describe a technically sound piece of scientific research with data that supports the conclusions. Experiments must have been conducted rigorously, with appropriate controls, replication, and sample sizes. The conclusions must be drawn appropriately based on the data presented.

Reviewer #1: Yes

Reviewer #2: Yes

We thank reviewers for these responses.


Question 3. Has the statistical analysis been performed appropriately and rigorously?

Reviewer #1: Yes

Reviewer #2: Yes

We thank reviewers for these responses.


Question 4. Have the authors made all data underlying the findings in their manuscript fully available?

The PLOS Data policy requires authors to make all data underlying the findings described in their manuscript fully available without restriction, with rare exception (please refer to the Data Availability Statement in the manuscript PDF file). The data should be provided as part of the manuscript or its supporting information, or deposited to a public repository. For example, in addition to summary statistics, the data points behind means, medians and variance measures should be available. If there are restrictions on publicly sharing data—e.g. participant privacy or use of data from a third party—those must be specified.

Reviewer #1: Yes

Reviewer #2: Yes

We thank reviewers for these responses.


Question 5. Is the manuscript presented in an intelligible fashion and written in standard English?

PLOS ONE does not copyedit accepted manuscripts, so the language in submitted articles must be clear, correct, and unambiguous. Any typographical or grammatical errors should be corrected at revision, so please note any specific errors here.

Reviewer #1: Yes

Reviewer #2: Yes

We thank reviewers for these responses.


Question 6. Review Comments to the Author

Please use the space provided to explain your answers to the questions above. You may also include additional comments for the author, including concerns about dual publication, research ethics, or publication ethics. (Please upload your review as an attachment if it exceeds 20,000 characters)

Reviewer #1: I have reviewed the revised MN entitled "Orthostatic hypotension and neurocognitive disorders in older women: Results from the EPIDOS cohort study".
Many thanks to the authors, as all the revisions made are sufficient.

Reviewer #2: The manuscript was substantially revised.

We thank reviewers for these responses.


Comments of Reviewer:

Major comments
Reviewer #1: Adjusting for absolute SBP or DBP
Pulse pressure was adjusted in the regression model. However, the systolic, diastolic or mean BP should be at least adjusted instead for pulse pressure. These results should be provided.

We thank Reviewer #1 for pointing out this important point.
We have taken this comment into account in the analysis of the results. By replacing, as proposed, Pulse Pressure (PP) by Mean Blood Pressure (MBP) while lying down at rest, in the analysis models, we do not observe any modification of the results.
These data from Table 2 are presented below but have not been added to the manuscript for clarity. These results have simply been described in the Results section. “Using mean arterial pressure value instead of PP as a covariate did not alter our results (data not shown)”.
Please see correction page 11 line 223-224.


Table 2. Multiple logistic regressions showing the cross-sectional association of baseline cognitive impairment (dependent variable) with Δ SBP and Δ DBP (Model A), and OH (Model B) adjusted for potential confounders (n=2715) 
	Baseline cognitive impairment						
	Model A	Model B					
	OR	[95% CI]	P-value	OR	[95% CI]	P-value	
Age	1.06	1.04 ; 1.09	<0.001	1.06	1.03 ; 1.09	<0.001	
Body mass index (ref: <21 kg/m²)			0.471			0.448	
∞	[21-25[ : Normal	0.82	0.61 ; 1.11		0.82	0.60 ; 1.10		
∞	[25-30[: Overweight	0.93	0.69 ; 1.26		0.92	0.68 ; 1.24		
∞	≥30 : Obesity	0.77	0.49 ; 1.21		0.76	0.48 ; 1.19		
Number of comorbidities 	0.89	0.84 ; 0.95	<0.001	0.90	0.84 ; 0.95	<0.001	
Orthostatic hypotension	-	-	-	1.16	0.88 ; 1.55	0.316	
Δ SBP	1.02	1.00 ; 1.03	0.028	-	-	-	
Δ DBP	0.99	0.99 ; 1.00	0.347	-	-	-	
MBP	1.00	0.99 ; 1.01	0.668	1.00	0.99 ; 1.01	0.637	
History of stroke	1.51	0.90 ; 2.55	0.120	1.48	0.88 ; 2.49	0.142	
IADL disability (i.e. score <7/8)	2.37	1.88 ; 2.98	<0.001	2.38	1.89 ; 3.01	<0.001	
Regular physical activity	0.81	0.66 ; 1.01	0.063	0.82	0.66 ; 1.02	0.074	
Use psychoactive drugs	1.08	0.87 ; 1.34	0.493	1.08	0.87 ; 1.34	0.491	
Use vitamin D supplements	0.75	0.55 ; 1.03	0.076	0.75	0.55 ; 1.03	0.077	
DBP: diastolic blood pressure; IADL: Instrumental Activities of Daily Living score; SBP: systolic blood pressure;  DBP: diatolic blood pressure; MBP: Mean Blood Pressure; P-value significant (i.e. P<0.05) indicated in bold.							
							


Question 7. PLOS authors have the option to publish the peer review history of their article (what does this mean?). If published, this will include your full peer review and any attached files.

If you choose “no”, your identity will remain anonymous but your review may still be made public.

Do you want your identity to be public for this peer review? For information about this choice, including consent withdrawal, please see our Privacy Policy.

Reviewer #1: No

Reviewer #2: Yes: Michiaki Nagai

We thank reviewers for this second review of our manuscript.
